# The core genome multi-locus sequence typing of *Mycoplasma anserisalpingitidis*

**DOI:** 10.1186/s12864-020-06817-2

**Published:** 2020-06-15

**Authors:** Áron B. Kovács, Zsuzsa Kreizinger, Barbara Forró, Dénes Grózner, Alexa Mitter, Szilvia Marton, Krisztina Bali, Anna Sawicka, Grzegorz Tomczyk, Krisztián Bányai, Miklós Gyuranecz

**Affiliations:** 1 Institute for Veterinary Medical Research, Centre for Agricultural Research, Hungária krt 21, Budapest, H-1143 Hungary; 2 Department of Poultry Diseases, National Veterinary Research Institute, Aleja Partyzantow 57, 24-100 Pulawy, Poland; 3 Department of Microbiology and Infectious Diseases, University of Veterinary Medicine, H-1078 István utca 2, Budapest, Hungary

**Keywords:** cgMLST, chewBBACA, Genotyping, *Mycoplasma anserisalpingitidis*, Waterfowl, Whole genome sequencing

## Abstract

**Background:**

*Mycoplasma anserisalpingitidis* is a waterfowl pathogen that mainly infects geese, can cause significant economic losses and is present worldwide. With the advance of whole genome sequencing technologies, new methods are available for the researchers; one emerging methodology is the core genome Multi-Locus Sequence Typing (cgMLST). The core genome contains a high percentage of the coding DNA sequence (CDS) set of the studied strains. The cgMLST schemas are powerful genotyping tools allowing for the investigation of potential epidemics, and precise and reliable classification of the strains. Although whole genome sequences of *M. anserisalpingitidis* strains are available, to date, no cgMLST schema has been published for this species.

**Results:**

In this study, Illumina short reads of 81 *M. anserisalpingitidis* strains were used, including samples from Hungary, Poland, Sweden, and China. Draft genomes were assembled with the SPAdes software and analysed with the online available chewBBACA program. User made modifications in the program enabled analysis of mycoplasmas and provided similar results as the conventional SeqSphere+ software. The threshold of the presence of CDS in the strains was set to 93% due to the quality of the draft genomes, resulting in the most accurate and robust schema. Three hundred thirty-one CDSs constituted our cgMLST schema (representing 42,77% of the whole CDS set of *M. anserisalpingitidis* ATCC BAA-2147), and a Neighbor joining tree was created using the allelic profiles. The correlation was observed between the strains’ cgMLST profile and geographical origin; however, strains from the same integration but different locations also showed close relationship. Strains isolated from different tissue samples of the same animal revealed highly similar cgMLST profiles.

**Conclusions:**

The Neighbor joining tree from the cgMLST schema closely resembled the real-life spatial and temporal relationships of the strains. The incongruences between background data and the cgMLST profile in the strains from the same integration can be because of the higher probability of contacts between the flocks. This schema can help with the epidemiological investigation and can be used as a basis for further studies.

## Background

*Mycoplasma anserisalpingitidis* is a waterfowl pathogenic bacterium that was first isolated in 1983 in Hungary [1, 2]. It commonly occurs alongside *M. anseris*, *M. cloacale,* and *M. anatis* and is present all over the world. It can be isolated from geese and occasionally from ducks [3]. This species can be transmitted both vertically and horizontally [4, 5]. The pathogen can cause significant economic losses [6–8]. Symptoms can include phallus and cloaca inflammation, egg infertility, airsacculitis, salpingitis, peritonitis, and embryo lethality [4–6]. In recent years the investigation of these bacteria have become a research priority [9]. In 2019 a species-specific PCR system was designed [3] that led to improved laboratory diagnosis. No vaccine is available for this mycoplasma as of yet, but antibiotics are used routinely to treat the infection [10]. For these reasons, the epidemiological monitoring and study of *M. anserisalpingitidis* is of high importance.

Conventional MLST system [11, 12] is among the most widespread molecular typing method, which focuses on the examination of 5 to 7 house-keeping genes, that can be found in all investigated strains. While this small selection allows for the sequencing of the genes even in bacteria, from which whole genome sequences are not possible to acquire, it still lacks the discriminatory power of the gene-by-gene comparison methods. In contrast, the core genome (cg) MLST system uses all the coding sequences that can be found in the majority of the strains. This is the reason why cgMLST schemas have higher resolution and are less susceptible to deletions and other mutations in the genomes, providing a more robust analysis than single nucleotide polymorphisms (SNP) based methods. With the recent advances in whole genome sequencing and a significant reduction of per-base costs, it is now easier to determine quickly and reliably the whole genome sequence and with it have access to a wider array of data than before. There are a few commercial software packages that can be purchased for cgMLST studies, for example, Ridom SeqSphere+ [13] and Bionumerics [14]. In recent years two cgMLST schemas have been developed for avian mycoplasmas using one of these platforms [15, 16]. Besides, open-source options are available, such as chewBBACA, which has been successfully used in many cgMLST studies for a multitude of microorganisms since its release [17–19].

*Mycoplasma anserisalpingitidis* has only recently been fully characterized [20], and as of the writing of this study, no cgMLST schema exists for *M. anserisalpingitidis*. The aim of the present examination was the genetic characterisation of 81 *M. anserisalpingitidis* strains with a robust and reliable cgMLST schema created in this study.

## Results

### Whole genome assembly

A total of 81 *M. anserisalpingitidis* whole genome sequences were used in this study, resulting in 81 multi fasta files with the average longest contig being 96,619 bp. Sequence reads of strains ATCC BAA-2147, MYCAV93, and MYCAV177 are available under the BioProject number: PRJNA554588, PRJNA553666, PRJNA554567 respectively. The raw nucleotide sequence reads of the further 78 *M. anserisalpingitidis* were uploaded to the Sequence Read Archive (SRA) of NCBI under the BioProject numbers: PRJNA602215, PRJNA602206, and PRJNA603657.

### Development of *M. anserisalpingitidis* cgMLST

#### cgMLST schema selection and evaluation

Using the modified chewBBACA algorithm a total of 1272 loci were selected for the wgMLST schema. Out of these loci, four cgMLST schemas were extracted based on different CDS presence thresholds (100, 95, 93, and 90% presence). Out of the examined four CDS presence thresholds, the 93% (including 331 CDSs) one resembled the metadata the most. The 331 loci represent approximately 42.77% of the whole CDS set of *M. anserisalpingitidis* ATCC BAA-2147. The most conservative allele showed six variants (MYCAV429-protein75), while 67 different genotypes were determined on the most variable allele (MYCAV429-protein17) among the 81 *M. anserisalpingitidis* strains.

**Fig. 1:**
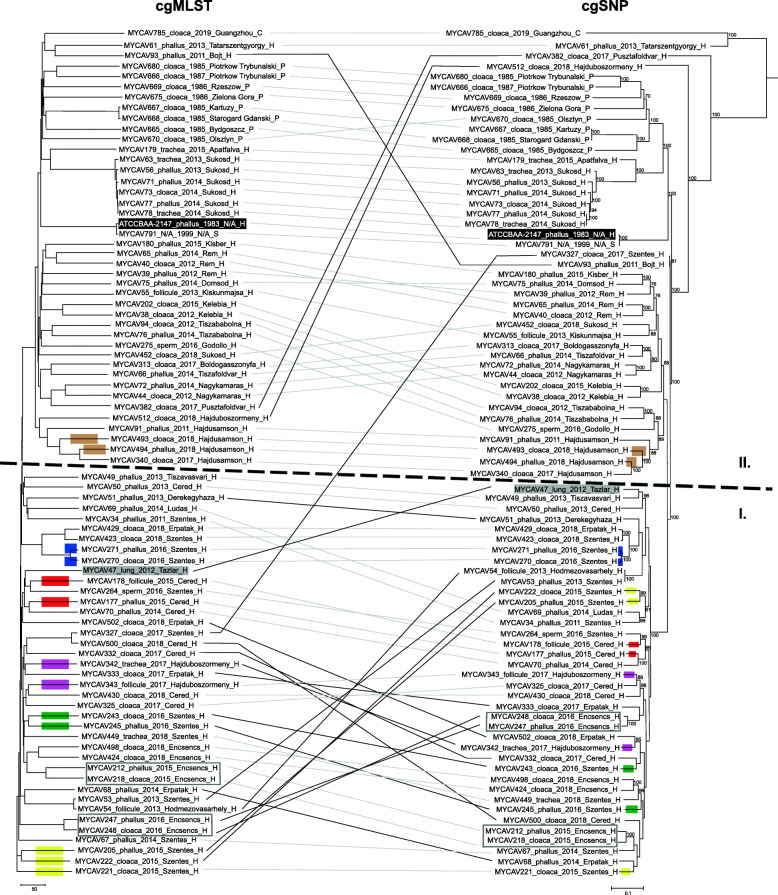
cgMLST and cgSNP based phylogenetic trees of 81 *M. anserisalpingitidis* strains. Strain ID, tissue sampled, year of isolation, location of origin, and country of origin are given for each strain. Abbreviations: lung – lung and air sac, phallus – phallus lymph, C – China, H – Hungary, P – Poland, S – Sweden, N/A – not available. The *M. anserisalpingitidis* type strain ATCC BAA-2147 is highlighted in black. Strains originating from the same animal are in grey rectangles. Coloured rectangles on the branches indicate that the strains originated from the same flocks, each colour represents a separate flock. Identical strains with similar or notably different topologies on the two dendrograms are bound with grey or black lines, respectively. **cgMLST.** Neighbor joining tree based on *M. anserisalpingitidis* cgMLST allelic profiles determined by the modified chewBACCA software. The dendrogram was created with GrapeTree software version 1.5.0 [21] **cgSNP.** Maximum Likelihood tree based on core genome SNP analysis. The dendrogram was created using the General Time Reversible model with Gamma distribution and bootstrap set to 500 iterations in MEGA-X version 10.0.5 [22]. Bootstrap values ≥70 are presented

Phylogenetic trees created from the initial wgMLST schema (data not shown) and the one from the final cgMLST schema showed similar topologies. Strains originating from the known same integration (Supplementary Table 1) showed close relationship and belonged to cluster I, while strains from other integrations and countries formed cluster II (Fig. 1).

In cluster I branch lengths between the subclades were notably shorter, than in cluster II, and cgMLST profiles did not correspond to geographical origins; however, subclades were formed by strains isolated mostly in the same year or in following years (Fig. 1). It should be noted, that three strains were included in cluster I, which originated from farms that did not belong to the integration forming the cluster. These strains were MYCAV47, the strain isolated from a duck, originating from Tazlar, approximately 80 km far from the closest farm (Szentes) of the integration; MYCAV49, a strain from Tiszavasvari, approximately 50 km far from the closest farm (Erpatak) of the integration and MYCAV69, a strain from Ludas, approximately 70 km far from the closest farm (Cered) of the integration.

A correlation was observed between the strains’ cgMLST profile and geographical origin in cluster II, in most cases strains from the same country (i.e. Poland) or same farms formed separate subclades (strains from Hungary, e.g. MYCAV91, MYCAV340, MYCAV493, and MYCAV494 are from the same city while MYCAV512 is from a city only approximately 20 km away). Strains from Sukosd, Hungary (the farm with the highest number of isolated strains in cluster II; *n* = 7) showed a correlation between year of isolation and their cgMLST profile also, forming two separate subclades (MYCAV452 from 2018 and MYCAV56, 63, 71, 73, 77 and MYCAV78 from 2013 and 2014) (Fig. 1).

Strains isolated from different tissue samples of the same animal (MYCAV212 and MYCAV218; MYCAV247 and MYCAV248) revealed highly similar cgMLST profiles. The majority of samples from the same flock also appear on the same branch (e.g. MYCAV205, MYCAV221 and MYCAV222 or MYCAV243, MYCAV245). However, example for strains from the same flocks with greater genetic distance between them (MYCAV177 and MYCAV178, MYCAV342 and MYCAV343) was observed as well. In these cases, the strains originated from different tissue samples of the birds (Fig. 1).

#### Comparison of the developed cgMLST schema with core genome SNP based analysis

The DNASTAR LaserGene suite found 25,518 SNPs in the pseudo-genome. The Maximum Likelihood phylogenetic tree based on the SNPs of the cgMLST CDS set and the Neighbor Joining tree from the cgMLST schema gave highly similar results. The three strain that originated from farms that did not belong to the integration forming cluster I remained in cluster I with the cgSNP analysis as well. The cgSNP based method also clustered most of the samples from the same flocks or animals together (e.g. MYCAV212 and MYCAV218, MYCAV245 and MYCAV243 or MYCAV 248 and 247); however, in certain cases the cgSNP based method was less sensitive (e.g. MYCAV205, MYCAV221 and MYCAV222 or MYCAV243 and MYCAV245). Notable differences were mainly observed in the topology of cluster I, and one strain (MYCAV327) belonging to the integration from where strains in cluster I originated was placed in cluster II in the cgSNP based dendrogram (Fig. 1).

## Discussion

*Mycoplasma anserisalpingitidis* is a common waterfowl pathogen present worldwide [2, 23]. Despite the economic losses it can cause, there are still major gaps in our knowledge about this microorganism. There is no vaccine for *M. anserisalpingitidis* as of yet and mainly antibiotic therapy can be used for the treatment of infected flocks and the fact that antibiotic resistance shows a growing trend among pathogenic microbes [10], warrants the inspection and monitoring of this bacterium. As a molecular biology based monitoring method, the cgMLST focuses on a large selection of CDSs found in most of the strains in a study, allowing high discriminatory power and making the system less susceptible to mutations, such as insertions and deletions.

The present study describes the first cgMLST schema for *M. anserisalpingitidis*. The open- source software BSR-Based Allele Calling Algorithm (chewBBACA) [24], adjusted with the consent of the original developers to be compatible with the mycoplasma genetic code (genetic code 4), was used to create the cgMLST schema. The modified algorithm provided highly similar results during the verification to previously published analyses on *M. gallisepticum* [15]*.* The developed *M. anserisalpingitidis* cgMLST assay constituted of 331 CDSs, 42.77% of the CDS set of *M. anserisalpingitidis* type strain ATCC BAA-2147. The CDSs in the schema were present in more than 93% of the strains in this study, which is a more lenient criterion than the default 100% in chewBBACA or the commonly used 95% [24], but it made for a more precise and robust assay. The suitability of the lower threshold value can be explained by the possible higher mutation rate of mycoplasmas [25, 26] or the quality of the sequencing and assembly. Deeper sequencing can improve the outcome, but it is important to note that our schema gave a precise result, which correlated with the epidemiological data, thus such deeper sequencing was not deemed necessary. Using this schema resulted in an assay that managed to differentiate between all of the strains in this study and group them into sub-clades that were continuous with the metadata of the samples.

The cgMLST analysis based genetic relations among the *M. anserisalpingitidis* strains were presented on Neighbor joining tree from the cgMLST profiles determined by chewBBACA, not on the sequences per se, which limits the usable topology metrics in setting up a phylogenetic tree. Nevertheless, the wgMLST and cgMLST based phylogenetic analyses showed high similarity, which indicates that the core genome schema is robust enough to be used as a basis of further studies, and the fact that the geographically and temporally close samples tend to be in the same groups further proves its precision.

The majority of the samples in this study originated from Hungary. While this fact restricts the scope of this assay, it also shows that the resolution of the method is exceptional, as in most cases even the strains with relatively small geographical distances are separated on the Neighbor joining tree. The fact that the strains from the same integration (cluster I, Fig. 1) showed higher variability and lacked correlation between genotype and geographical origin could indicate that the hosts were moved frequently or had contact with each other. This practice could hinder the defence against the bacterium as a potentially pathogen free stock could be infected or re-infected.

The results of the developed cgMLST schema and cgSNP based analysis were highly congruent, with notable differences in the topology observed mostly among the strains in cluster I (Fig. 1). The reason behind the observed differences can be the fact that the two software used for the analyses (chewBBACA and LaserGene) apply different approaches to find SNPs (CDS alignment and read mapping, respectively). It is also important to note that the cgSNP based phylogenetic tree was assessed with a different method (Maximum Likelihood) than the allelic profile based tree (Neighbor joining).

The developed cgMLST assay proved to be robust and showed high resolution, revealing high variability of the examined 81 *M. anserisalpingitidis* strains, and discriminating two main clusters among them congruent with the strains’ epidemiologic data (Fig. 1). The fact that the assay had to be preceded by time-consuming whole genome sequencing is greatly offset by the lack of primer designing and validating steps. Besides, as the target species is a relatively fast growing bacterium, with lower requirements during isolation compared to other *Mycoplasma* species, the presented cgMLST can be recommended for diagnostic use also.

The relatively high number of Hungarian samples indicates that the infection may be widespread in the Carpathian Basin, but the fact that there were samples from Poland, Sweden, and China suggests that the microorganism can be found all over the world. China is the world leader in goose production by a huge margin and it is especially important to further explore the extent of *M. anserisalpingitidis* infection there [27]. It is important to note that the lack of a substantial number of samples from different countries is likely because of the relatively small awareness of this particular pathogen instead of the lack of infection in waterfowl stocks. Our cgMLST assay allows for the precise typing of *M. anserisalpingitidis* strains, which can lead to a better understanding and defence against this pathogen.

## Conclusions

The presented cgMLST schema is the first published genotyping assay to differentiate between *M. anserisalpingitidis* strains from different integrations, geographical location, and time period. The high resolution of the developed cgMLST allows its use in phylogenetic analyses and diagnostics of potential outbreaks, and it can be the basis of future studies exploring the phenotypic differences of the strains in different subclades.

## Methods

### Strain preparation and whole genome assembly

A total of 78 *M. anserisalpingitidis* genomes were sequenced successfully in this study originating from Hungary (*n* = 68), Poland (*n* = 8), Sweden (*n* = 1) and China (n = 1). The detailed information of the strains can be found in Supplementary Table 1. DNA was extracted from the strains with the QIAamp DNA Mini kit (Qiagen, Inc., Hilden, Germany). Next-generation sequencing was performed on NextSeq 500 Illumina equipment (Illumina, Inc., San Diego, CA USA), with NextSeq 500/550 High Output Kit v2.5 reagent kit. Publicly available whole genome sequences of an additional three strains, ATCC BAA-2147, MYCAV93, and MYCAV177 [9] were involved also in the analyses, resulting in 81 strains altogether. The raw sequence reads were first quality checked with the FastQC software version 0.11.8 (downloaded on 2019.02.13) [28], and trimmed with the NxTrimm software version 0.4.3 (downloaded on 2019.02.13) [29] and paired with Geneious Prime version 2019.2.1 (downloaded on 2018.11.12) [30]. The draft genomes were assembled with the SPAdes program version 3.13.0 (downloaded on 2019.03.13) [31].

### Adjustment and verification of chewBBACA software for mycoplasma genetic code compatibility

The BSR-Based Allele Calling Algorithm (chewBBACA) version 2.0.17 (downloaded on 2019.06.22) [24] was used to create a whole-genome (wg) and then a core genome (cg) schema. As of the writing of this article, chewBBACA is hard coded to only use the standard genetic code 11 which creates an obstacle in analysing any bacteria using genetic code 4 (the mycoplasma genetic code). For this reason, the source code had to be modified slightly to serve as an adequate framework for mycoplasmas. The only difference between the standard genetic code (11) and mycoplasma genetic code (4) is that the TGA codon codes Tryptophan (Trp) instead of a stop codon [32]. Hence, every instance in the chewBBACA source code referring to genetic code 11 was changed to genetic code 4, but the software was not tampered with besides this small change, which was done with the knowledge and approval of the original software developers. The chewBBACCA software makes use of the Prodigal program version 2.6.0 (downloaded on 2019.06.22) or above [33] for finding Coding DNA Sequence (CDS) in the whole or draft genomes and BLAST+ version 2.9.0 (downloaded on 2019.06.22) [34] for comparison between the alleles.

As a first step in the verification of the modified software, data from the whole genome fasta file of *M. anserisalpingitidis* ATCC BAA-2147 annotated with Prodigal were compared to the NCBI annotated genome. The Prodigal managed to find all of the CDSs in the sequence with the exception of 11.

The applicability of the modified software was further checked using a previously developed *M. gallisepticum* cgMLST schema [15] as a comparison. The sequence reads of *M. gallisepticum* strains from BioProject PRJNA401291 were downloaded and assembled into draft genomes. The 55 assembled draft genomes were then analysed with chewBBACA as described below. The allelic profiles of the Ridom SeqSphere+ and chewBACCA cgMLST schemas (Supplementary Table 2) were used in the creation of two Neighbor joining trees with GrapeTree software version 1.5.0 (downloaded on 2020.04.) [21] using the FastME implementation [35] as seen in Supplementary Fig. 1.

### Development of *M. anserisalpingitidis* cgMLST

The creation of the *M. anserisalpingitidis* cgMLST schema was done with the modified chewBBACA version 2.0.17 [24]. First, a wgMLST schema was set up containing every CDS, and then the paralogous alleles were filtered, thus creating the cgMLST schema.

### Creation of wgMLST

The wgMLST schema of *M. anserisalpingitidis* was created with the modified chewBBACA software [24]. A training file was created in Prodigal [33] with *M. anserisalpingitidis* ATCC BAA-2147 and this file was used in the further steps of the study. The wgMLST schema from the 81 *M. anserisalpingitidis* strains was assessed first with the CreateSchema operation, during which every genome is annotated one by one. Sequences are compared in a pairwise fashion and an all-against-all BLASTP search and the BLAST score ratio (BSR) is calculated [36]. This allows for the genes that are coding the same or very similar (by default BSR above 0.6) proteins to be considered alleles of the same locus and to be collected in a database. The second step of the wgMLST creation is the AlleleCall operation during which the software populates the schema and an algorithm detects whether a CDS can correspond to more than one locus and a BLASTP database is created from all the translated CDSs. This database can be used as a base for the BLASTP search, during which the sequence can be classified either as a new locus or as a missing one.

### Extraction of the cgMLST schema

The quality of the wgMLST schema was tested with the TestGenomeQuality operation. During the quality test, it was found that the majority of the CDSs were not present in all genomes. Four cgMLST schemas were extracted from the wgMLST schema with the ExtractCgMLST operation of the modified chewBBACA software, with CDS presence thresholds 100, 95, 93, and 90%. Neighbor-joining trees were created from the extracted allelic profiles in Phyloviz software version 2.0 (downloaded on 2019.09.12) using Hamming distance and Saitou - Nei criterion [37, 38] (data not shown). With the 100% presence rate, the schema consisted of only 73 CDSs, while 291 with 95%, 331 with 93% and 408 CDSs were present with the 90% threshold. The results of the four cgMLST schemas were manually examined and the one best representing the metadata of the strains was chosen. The best result was achieved with a 93% threshold with 331 CDSs that were present in the schema, constituting of approximately 42.77% of the CDS set of *M. anserisalpingitidis* ATCC BAA-2147.

### Comparison of the developed cgMLST schema with core genome SNP based analysis

The CDSs constituting our cgMLST schema were extracted from the strain MYCAV93, randomly chosen out of the available whole genomes on NCBI. These CDSs were concatenated, creating a pseudo-genome. The sequence reads of the 80 strains (all examined strains except MYCAV93) were mapped with DNASTAR LaserGene suite version 17.0.2.1 (downloaded on 2020.04.20) [39]. The SNPs were filtered and an alignment was created from the ones corresponding to the same position. The alignment was analysed with MEGA-X version 10.0.5. (downloaded on 2019.02.25) [22] and a Maximum Likelihood tree was created using the General Time Reversible model with Gamma distribution and bootstrap set to 500 iterations. The cgSNP analysis based phylogenetic tree was compared with the Neighbor joining tree based on cgMLST profiles assessed by GrapeTree software version 1.5.0 (Fig. 1).

## Supplementary information

**Additional file 1: Supplementary Table 1.** Background information and cgMLST profiles of the 81 *M. anserisalpingitidis* strains examined in the study.

**Additional file 2: Supplementary Table 2.** chewBBACA cgMLST profiles of 55 *M. gallisepticum* strains analysed with Ridom SeqSphere+ in a previous study. The cgMLST profiles of *M. gallisepticum* strains from the publication of Ghanem et al. [15] were determined with the in-house adjusted chewBBACA software [24], compatible with the mycoplasma genetic code.

**Additional file 3: Supplementary Figure 1.** Comparison between two *M. gallisepticum* phylogenetic trees using the Ridom SeqSphere+ and chewBBACA cgMLST schemas. Neighbor joining trees were created with GrapeTree software version 1.5.0 [21]. Identical strains with similar or notably different topologies on the two dendrograms are bound with grey or black lines, respectively. A. Phylogenetic tree created based on *M. gallisepticum* cgMLST allelic profiles determined by Ridom SeqSphere+ software [15]. B. Phylogenetic tree created based on *M. gallisepticum* cgMLST allelic profiles determined by the modified chewBACCA software.

## Data Availability

All data generated or analysed during this study are included in this published article. The sequence reads of the strains have been uploaded to the Sequence Read Archive (SRA) under: PRJNA554588, PRJNA553666, PRJNA554567, PRJNA602215, PRJNA602206, and PRJNA603657. The cgMLST schema can be found under: 10.6084/m9.figshare.12129054

## Abbreviations

MLST: Multi-Locus Sequence Typing
wgMLST: Whole genome Multi-Locus Sequence Typing
cgMLST: Core genome Multi-Locus Sequence Typing
CDS: Coding DNA sequence
chewBBACA: BSR-Based Allele Calling Algorithm
NCBI: National Center for Biotechnology Information
BSR: BLAST score ratio
SRA: Sequence Read Archive
