# Supplementary figures and images for: The core genome multi-locus sequence typing of Mycoplasma anserisalpingitidis
Source: BMC Genomics. 2020 Jun 15;21:403. doi: 10.1186/s12864-020-06817-2 (PMC7296915; doi:10.1186/s12864-020-06817-2)

A

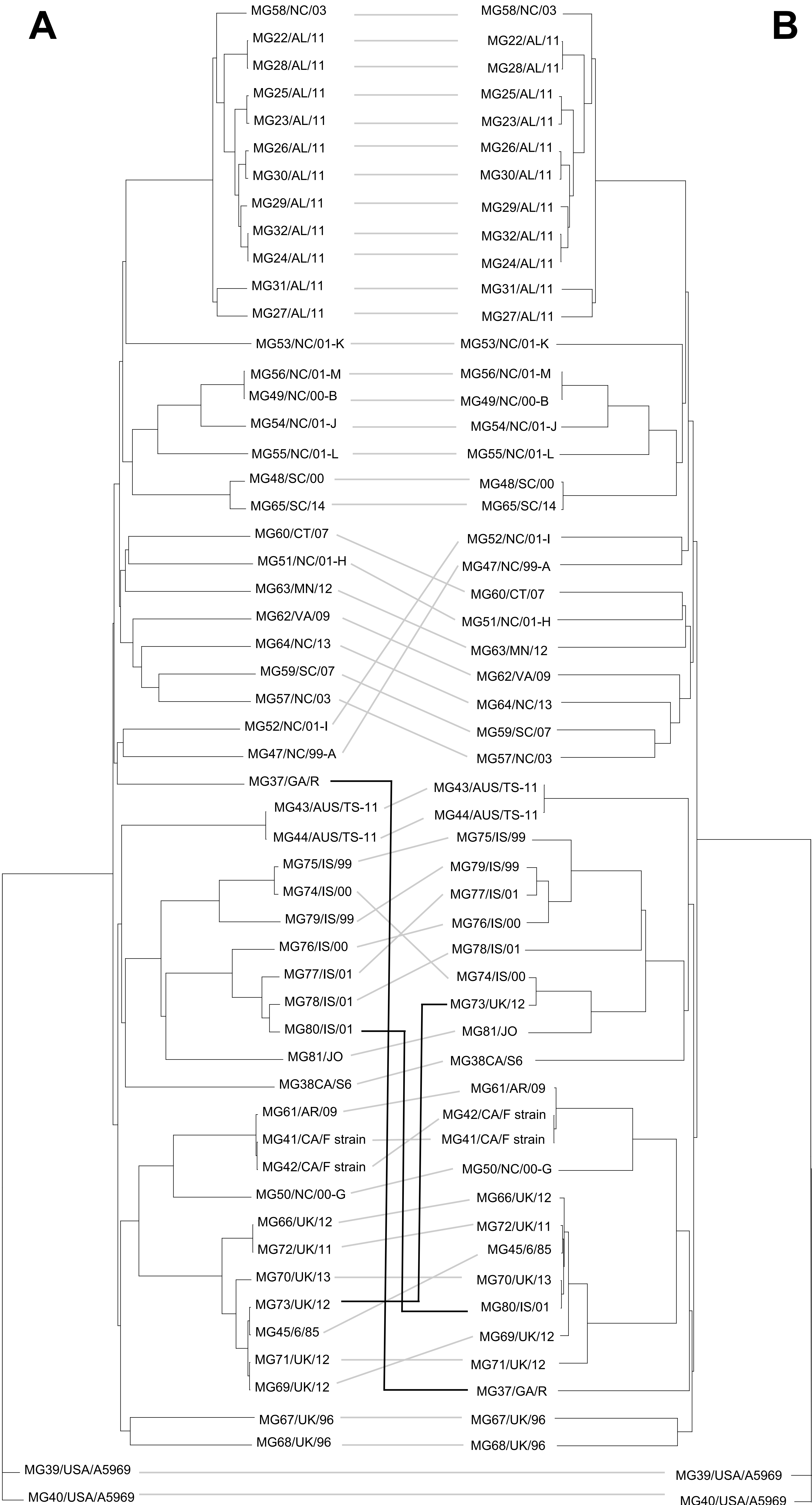

B

Supplement: Supplementary file 3 — Additional file 3: Supplementary Figure 1. Comparison between two M. gallisepticum phylogenetic trees using the Ridom SeqSphere+ and chewBBACA cgMLST schemas. Neighbor joining trees were created with GrapeTree software version 1.5.0 [21]. Identical strains with similar or notably different topologies on the two dendrograms are bound with grey or black lines, respectively. A. Phylogenetic tree created based on M. gallisepticum cgMLST allelic profiles determined by Ridom SeqSphere+ software [15]. B. Phylogenetic tree created based on M. gallisepticum cgMLST allelic profiles determined by the modified chewBACCA software. [file 12864_2020_6817_MOESM3_ESM.pdf]
